# The Quantitative Genetic Architecture of the Bold-Shy Continuum in Zebrafish, *Danio rerio*

**DOI:** 10.1371/journal.pone.0068828

**Published:** 2013-07-01

**Authors:** Mary E. Oswald, Mathew Singer, Barrie D. Robison

**Affiliations:** Department of Biological Sciences, University of Idaho, Moscow, Idaho, United States of America; Tulane University Medical School, United States of America

## Abstract

In studies of consistent individual differences (personality) along the bold-shy continuum, a pattern of behavioral correlations frequently emerges: individuals towards the bold end of the continuum are more likely to utilize risky habitat, approach potential predators, and feed under risky conditions. Here, we address the hypothesis that observed phenotypic correlations among component behaviors of the bold-shy continuum are a result of underlying genetic correlations (quantitative genetic architecture). We used a replicated three-generation pedigree of zebrafish (*Danio rerio*) to study three putative components of the bold-shy continuum: horizontal position, swim level, and feeding latency. We detected significant narrow-sense heritabilities as well as significant genetic and phenotypic correlations among all three behaviors, such that fish selected for swimming at the front of the tank swam closer to the observer, swam higher in the water column, and fed more quickly than fish selected for swimming at the back of the tank. Further, the lines varied in their initial open field behavior (swim level and activity level). The quantitative genetic architecture of the bold-shy continuum indicates that the multivariate behavioral phenotype characteristic of a “bold” personality type may be a result of correlated evolution via underlying genetic correlations.

## Introduction

Until recently, individual variation in behavior among animals was disregarded as ‘noise’. However, it is becoming clear that animals across the taxonomic spectrum exhibit consistent individual differences in behavior (personality), although the mechanisms underlying the evolution and persistence of this variation are unclear. One of the most well studied axes of behavior in this regard is the bold–shy continuum, which describes an animal’s propensity to take risks [1,2]. Boldness studies therefore typically quantify individual responses to risky situations but the specific assays and contexts can vary dramatically between studies. While the disparate methods used to quantify boldness can hinder direct comparisons across studies [3], when multiple behavioral assays are employed in a single study, a consistent pattern of behavioral correlations frequently emerges: individuals towards the bold end of the continuum are more likely to utilize risky habitat, approach potential predators, and feed under risky conditions [1,2,4,5].

How do these complex correlated sets of behaviors evolve? There are two primary schools of thought [4]: one, that behavior is constrained to evolve in the observed correlated fashion due to underlying genetic correlations; two, that the behavioral correlations are a byproduct of correlated selection occurring within a given adaptive landscape (and thus may be decoupled under a different landscape). However, studies of the underlying genetic basis of these phenotypic correlations are lacking. One of the most direct ways to differentiate among these hypotheses is to estimate the G matrix of the behavioral components of the bold-shy continuum. A G matrix describes the quantitative genetic architecture for a series of traits by estimating their additive genetic variances and covariances. Because genetic covariance among traits can constrain evolution, the G matrix can also be used to calculate “evolutionary lines of least resistance” – patterns of correlated evolution that are facilitated by the genetic variance covariance structure [5]. The G matrix can also be used to calculate heritabilities and genetic correlations. Heritability estimates exist for some single component behaviors of the bold-shy continuum [6], but estimates for genetic correlations among the behaviors are limited [7]. Most of the recent work on the genetic basis of personality has focused on behavioral syndromes, or correlations across multiple axes of personality, such as boldness-aggression [8] or boldness-exploratory behavior [9], rather than the quantitative genetic architecture that underlies a particular personality axis.

Here, we use the zebrafish to test the hypothesis that observed phenotypic correlations among component behaviors of the bold-shy continuum are a result of underlying genetic correlations. We performed a three-generation replicated selection experiment to test three predictions of our hypothesis: 1) selection on one component behavior would result in changes to other component behaviors, 2) estimates of the G-matrix would reveal significant genetic correlations among these component behaviors, and 3) the predominant axis of genetic variation within the G matrix should align with the bold-shy continuum. Our findings confirm our predictions and support the hypothesis that evolution along the multivariate bold-shy continuum may be, at least in part, driven by underlying quantitative genetic architecture.

## Methods

### Ethics Statement

Experimental fish were generated from adult zebrafish of the Scientific Hatcheries (SH) strain that had been bred and maintained in our Aquaneering Inc (San Diego, USA) recirculating zebrafish facility. Fish were maintained at 28.5°C on a 14 hours light: 10 hours dark photoperiod and fed twice daily with flake food and live brine shrimp. The University of Idaho Animal Care and Use Committee approved all aspects of care and observation in this study under protocol 2008-49.

### Selection design

We designed a selection experiment in which we created lines of “bold” and “shy” zebrafish, capitalizing on detailed pedigree data to study the quantitative genetic architecture of multiple component behaviors of the bold-shy continuum. Although the zebrafish has long been a model for developmental research, recent work has demonstrated its suitability for both behavior and genetics research [10–14]. In particular, behavioral variation (including boldness) among individuals and populations of zebrafish is well documented [11,12,15–18].

First, randomly-chosen adult zebrafish (Scientific Hatcheries strain) from our general population were placed in individual tanks and assayed for three behaviors (horizontal position, swim level, and feeding latency, described below under Behavioral Assays) in October of 2006. We calculated repeatability [19] for each behavior, and the behavior with the highest repeatability (horizontal position, repeatability = 0.56) was chosen as the selection criterion. To create the selected lines, fish from the behavioral assays were ranked according to their average horizontal position. The five highest scored males and females were combined into breeding pairs to propagate the High line (predicted to be the boldest), while the five lowest scored males and females were paired to create the Low line (predicted to be the shyest). Pairs were maintained in 2.8L tanks within the zebrafish facility, assigned a numeric family identifier, and provided with an opportunity to breed in our standard breeding cassettes (placed directly into the tank, with mesh to contain the eggs and prevent parental cannibalism) once per week for 10 weeks in order to generate enough adult animals from each family.

Eggs were collected after each breeding opportunity, reared in beakers on a diet of *Paramecia* and *Artemia*, then transferred to tanks within the zebrafish facility and maintained on the standard facility’s feeding schedule. At three months of age, fish (Generation 1) were placed in individual tanks for behavioral typing. Again, the five males and five females with the highest average horizontal position were paired within the High line, and the five males and five females with the lowest average horizontal position were paired within the Low line. Pairs were bred, and the progeny (Generation 2) were reared, placed in individual tanks, and behavior-typed, resulting in a detailed pedigree for the three generations (N=301).

This selection experiment was then repeated beginning in June of 2010, starting again with randomly-chosen adult SH fish from the general population, but using a different observer. Breeders were again selected based on horizontal position, and progeny reared through Generation 2 (N=398). All fish in the experiment were given unique identification numbers and detailed records were kept as to the identity of all breeders and their progeny. Numbers of fish per replicate and behavior assays for each generation are provided in Table 1, sample sizes by sex are provided in Table S1.

**Table 1:** Sample size and behavioral assays used in each generation of zebrafish breeding.

		**Generation**		
**Replicate**		**0**	**1**	**2**
1	Total N	76	57	173
	N High line	-	30	103
	N Low line	-	27	70
2	Total N	78	180	180
	N High line	-	88	92
	N Low line	-	91	89
	Behaviors	HP, SL, FL	HP, SL, FL	HP, SL, FL, OF

Note. Behavior abbreviations: HP = Horizontal Position, SL = Swim Level, FL = Feeding Latency, OF = Open Field, and ST = Shoaling Tendency.

### Behavioral assays

#### Place preference

Adult zebrafish were randomly placed in individual 1L tanks (5 cm wide x 25 cm deep x 12 cm water height) within the top row of the zebrafish facility to minimize disturbances and reduce variation in light exposure. Individuals were visually isolated using white paper barriers between the tanks and allowed to habituate to their tanks for one week [15]. Tanks were assigned a numeric identifier so that subsequent observations were not influenced by knowledge of family or line identification.

The first behavioral assay quantified vertical and horizontal place preference of the fish within the tank relative to the observer. We specifically chose to measure these place preferences in the presence of the observer (rather than have the observer behind a blind) to include the possibility of variation in fearfulness towards humans among the fish. Horizontal preference (horizontal position) was recorded as a binary variable, with ‘1’ indicating the fish was within one body-length of the front of the tank (next to the observer), and ‘0’ indicating the fish was farther away than one body-length [15,20]. This behavior indicates the willingness of the fish to approach the observer, similar to the ‘confidence’ behavior used in studies of fox domestication [21]. Vertical preference (swim level) was recorded as a categorical variable using rows of string across the front of the tanks, dividing each tank into six vertical sections (each 2cm tall for a total depth of 12cm). Increased swim level (swimming near the surface) has been associated with reduced fearfulness and predator avoidance in hatchery fishes [22] and found to differ among zebrafish strains [12,20]. The observer stood 0.5m from the front of the tank and at one second intervals, for a total of three seconds, recorded the horizontal and vertical locations of the fish in the tank. This assay was conducted twice per day (approximately 9: 00am and 2: 00pm) for four days, resulting in six observations per fish per day for each of the two place preferences (24 total observations per fish per behavior).

#### Feeding latency

The second behavioral assay measured the length of time in seconds that fish took to begin feeding from the surface of the water when provided their morning meal (feeding latency). This assay has previously revealed strain variation in our laboratory [16] and can be influenced by hunger and perceived risk [16,23]. Feeding latency was measured just after the morning place preference assay for each of the four observation days. Following normal feeding protocols for the facility, the observer stood 0.5m from the tank and provided flake food (Tetramin flake food; 8mg ± 2mg per feeding event) by hand through a hole in the tank lid. A stopwatch was used to quantify the time fish took to obtain the first piece of food from the surface. The surface was chosen as captive fish populations are generally fed from the surface and the surface of the water is often associated with increased risk of predation [22,24–27]. Flake food was chosen for its propensity to float, although as time progressed (greater than 30sec) the food would tend to sink lower into the water column. Fish that foraged from within the water column were recorded, but the stop-watch continued until the fish foraged from the surface. Any fish that had not fed from the surface within three minutes was given a feeding latency value of 180sec and observation was discontinued until the next day. All fish were typically assayed within 30min of observing the first fish. All fish were given a second feeding (with no behavioral observations) after the 2:00pm place preference assay.

#### Open field

As exploratory behavior and response to novelty [1] are often associated with boldness, we also tested the selected lines for their behavior in a novel open-field [8,28–30]. Fish were provided with a novel environment (a 19L glass aquaria with overhead cover) and scored for their behavior at two time points to determine whether selection for horizontal position would influence behavior in a different context. We predicted that behavior in an open field would be related to boldness, such that bold fish would be more exploratory of the new environment.

The aquarium measured 40.6 cm wide x 21.6 cm deep x 22.8 cm water height and the back was marked with a 3x3 grid (each cell measured 7.6 x 13.5 cm) to facilitate tracking and one-third of the top of the tank was covered with green mesh as cover [27]. The grid layout was similar to the number pad of a computer, such that as the fish moved across gridlines, the observer pushed a number on the pad corresponding to the grid cell entered by the fish. A custom-designed computer program created in our laboratory (TimeStamper) recorded the number pushed and the time, compiling the data into a proportion of time spent in each of the nine grid cells. In addition, the program totaled numbers pushed for an estimate of activity level and depression of another key recorded the amount of time in seconds that a fish spent frozen motionless. The tanks for this assay were located in a separate room from the main facility, with visual barriers surrounding the back and two sides of the tank to minimize environmental effects on behavior. The observer stood behind a blind in front of the tank to collect behavioral data.

This assay was conducted on a subset of randomly chosen fish from Generation 2 of both replicates (replicate 1: N_total_=74, N_High_ =36, N_Low_ =38; replicate 2: N_total_=66, N_High_ =36, N_Low_ =30) starting two weeks after the place preference and feeding latency assays were completed. Open field trials were conducted one hour post-feeding to minimize variation among subjects due to satiety. In this test, a fish was taken to the observation room, and netted from its 1L tank into the 19L tank. The observer immediately began manually tracking the fish’s activity from behind a blind in real time for a period of four minutes (initial period). The fish was then left in the tank for 56 minutes, after which the fish was again tracked for four minutes (post-acclimation period). At the end of the tracking period, the fish was netted out of the aquaria and returned to its 1L tank. This assay provided us with place preference (both swim level and horizontal space-use), use of cover, and activity level behaviors (time spent frozen motionless as well as the average number of cell changes) for the fish in an open-field environment, both upon initial contact with the environment and after a period of acclimation. For those fish that spent any amount of time frozen motionless (and thus not actively choosing a location), place preference and cover use were calculated as proportions using the time the fish was moving in the tank.

### Analyses

#### Behavior

To test for a response to selection (Prediction 1), horizontal position, swim level, and feeding latency were analyzed using a repeated-measures, mixed-model ANOVA in SAS 9.1 within each replicate and generation. For these analyses, daily averages for each behavior were calculated. The model included line, sex, family (block effect), and day of each behavioral assay (as a block effect and as a repeated measure with each fish as the subject). For feeding latency, significant deviations from normality in the model residuals were rectified by inverse-transformation of the data (thus higher values reflect more rapid feeding). Body size was excluded from the model after it was found to be non-significant for every behavior and to not differ between the lines, although females were larger than males (female mass - 656.5mg ± 8.3mg; male mass - 357.5 ± 7.9mg; female length - 31mm ± 0.14mm; male length - 27.8 ± 0.13mm).

With one exception, behaviors during the open field assay were analyzed using a similar mixed-model ANOVA, with line and sex coded as main effects in the model and family and replicate as a blocking effect. While duration of time spent frozen was analyzed using the above ANOVA model (with significant deviations from normality in model residuals rectified using arc-sin transformations), testing whether the lines differed in the proportion of individuals that froze was performed using a chi-squared test.

#### G-matrix

To test for genetic correlations among the behaviors (Prediction 2), the G-matrix was estimated using the restricted maximum likelihood program WOMBAT [31]. Narrow-sense heritability, genetic correlations, and phenotypic correlations were quantified for horizontal position, swim level, and feeding latency (the three behaviors with the most detailed pedigree information). Lack of open field data from the parental generation and Generation 1 precluded estimation of heritabilities and genetic correlations for these traits. Because all of our measures were bounded on both ends, the data were normalized using an arcsin-square root transformation and then standardized to a mean of 0 and standard deviation of 1. Our animal model [8,32] included sex and replicate as fixed effects, animal as a random effect and there were 630 individuals total in the pedigree. Confidence intervals (95%) around the point estimates of narrow-sense heritability and genetic correlation were calculated using the standard errors of each parameter.

Our hypothesis that the bold-shy continuum consists of a set of genetically correlated component behaviors suggests that the predominant axis of genetic variation should align with the bold-shy continuum (Prediction 3). We therefore analyzed the G-matrix for dimensionality [33] and angles between the first principle component, Gmax, and the selection gradient were calculated separately for the bold and the shy lines [5,34]. To calculate the angles, a fitness value of 1 was assigned to individuals that contributed to the next generation of that particular line, and a value of 0 was given to the remainder. The selection gradients, β, were calculated using logistic regression [35].

## Results

### Response to Selection

**Figure 1:**
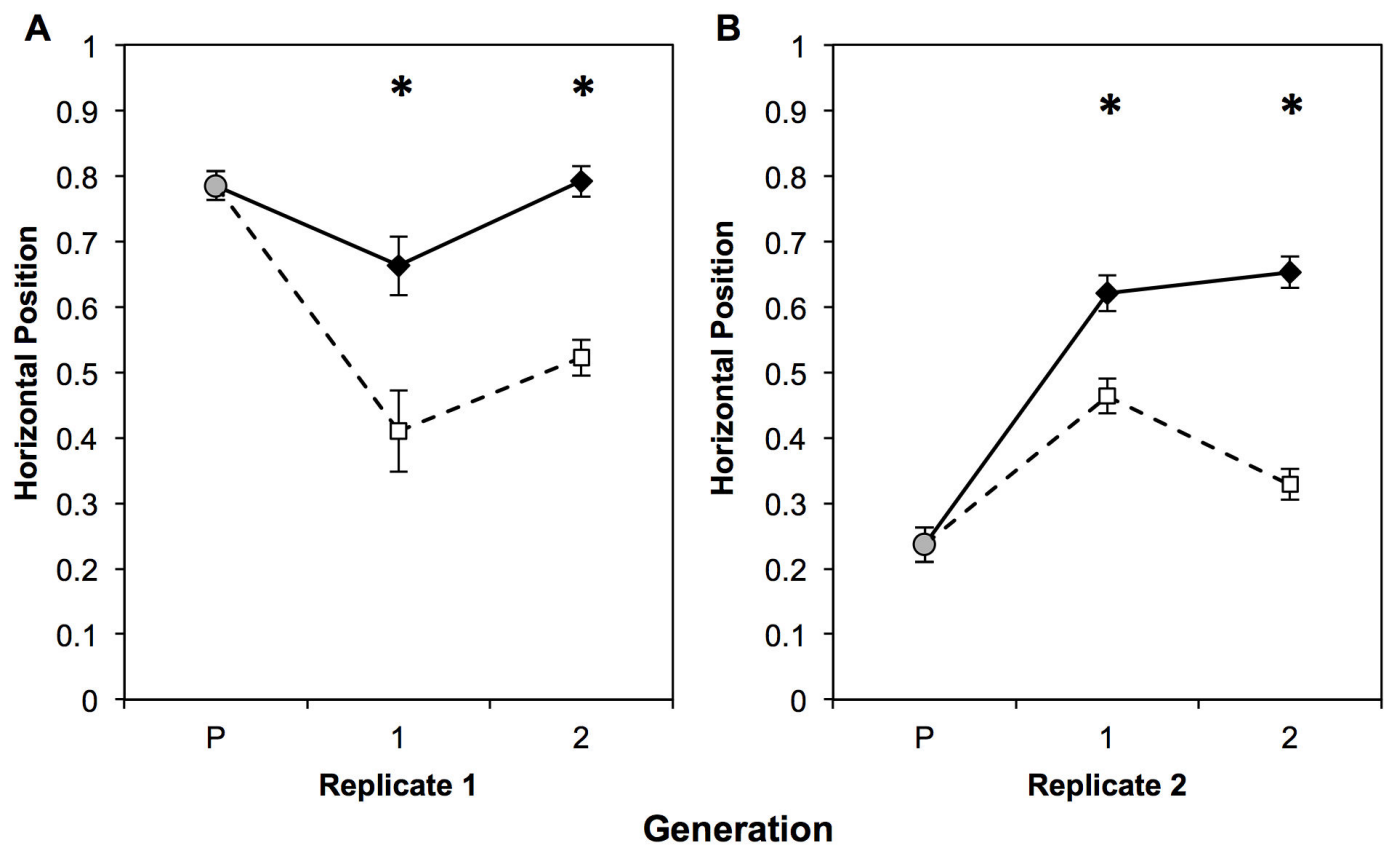
Response to two generations of selection on horizontal position (time near observer) in zebrafish. A: Replicate 1, B: Replicate 2. x-axis generations: P = unselected parental generation (gray circle), 1 = first generation progeny from selected parents, 2 = second generation progeny from selected first generation parents. Black diamonds with a solid line represent means ± SE of High line fish, open squares with a dashed line represent means ± SE of Low line fish. “*” indicate significant (p<0.05) differences between lines within a generation using a mixed-model ANOVA.

Across both replicates, we detected a significant response to selection in just one generation such that progeny from high horizontal position parents had a higher mean horizontal position than fish with low horizontal position parents (Figure 1; replicate 1 F_1,46_ = 21.24, *P*<0.0001; replicate 2 F_1,168_ = 44.8, *P*<0.0001). This line effect was maintained in the second generation of selected progeny (replicate 1 F_1,163_ = 158.42, *P*<0.0001; replicate 2 F_1,166_ = 253.1, *P*<0.0001). Across both replicates and generations, our mixed models also revealed significant differences between the sexes in their horizontal position, with females scoring higher than males for this behavior (*P*<0.04 for all replicate/generation combinations). Selection differentials (the average differences between individuals selected as breeders and their entire generation) were: Parental breeds 0.16 (High), 0.35 (Low); Generation 1 breeds 0.28 (High), 0.31 (Low); and Generation 2 breeds 0.21 (High), 0.31 (Low). The frequency distributions of horizontal position across all generations and replicates are provided in Figure S1.

### Prediction 1: Correlated Responses to Selection

#### Swim Level

**Figure 2:**
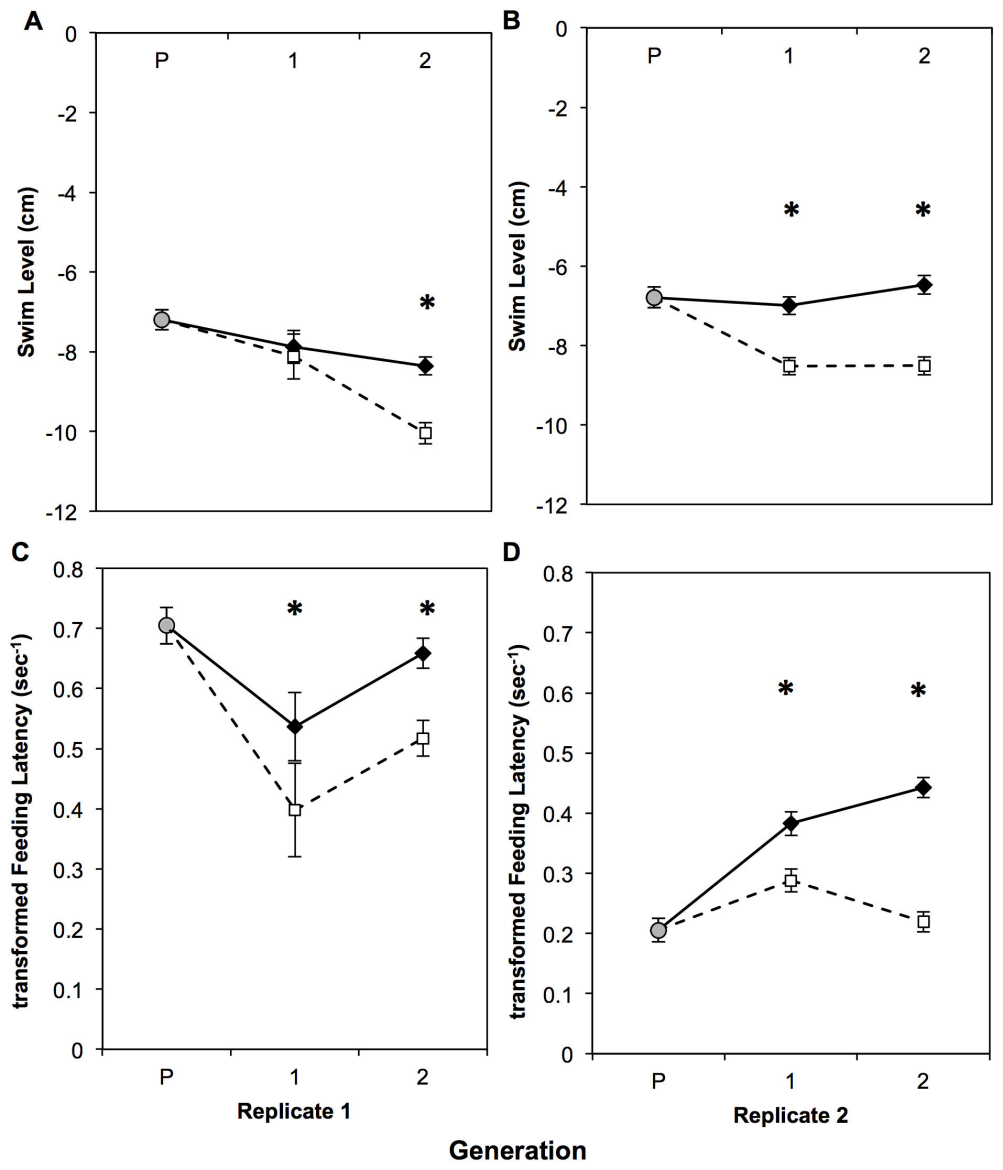
Response of Swim Level and Feeding Latency to selection on horizontal position in zebrafish. Replicate 1 (A & C), Replicate 2 (B & D). P = unselected parental generation (gray circle), 1 = first generation progeny from selected parents, 2 = second generation progeny from selected parents. Black diamonds with a solid line represent means ± SE of High line fish, open squares with a dashed line represent means ± SE of Low line fish. “*” indicate significant (p<0.05) differences between lines within a generation using a mixed-model ANOVA.

In replicate 1, there was no divergence between lines in the first generation for swim level (*F*_1,47_ =0.27, *P*=0.6035). By the second generation, however, swim level showed a significant effect of line (*F*_1,163_ = 58.84, *P*<0.0001), where fish from the Low line swam lower in the water column than High line fish (Figure 2a). We also detected a main effect of sex in the second generation (*F*_1,163_ = 41.2, *P*<0.0001), where males swam lower in the water column than females.

In replicate 2, swim level diverged within the first generation, again with Low line fish swimming lower in the water column than High line fish (Figure 2b; *F*_1,168_ = 61.09, *P*<0.0001). Also, males were significantly lower in the water column than females (*F*_1,168_ = 16.45, *P*<0.0001). Replicate 2 Generation 2 continued that same pattern of divergence between lines (*F*_1,166_ = 107.74, *P*<0.0001), but there was no effect of sex (*P*>0.05). The frequency distributions of swim level across all generations and replicates are provided in Figure S2.

#### Feeding Latency

Feeding latency diverged among the lines with the first generation for both replicates (replicate 1 F_1,47_ = 7.72, *P*=0.0078; replicate 2 F_1,168_ = 55.43*P*<0.0001). For this behavior, fish from the Low line took significantly longer to begin foraging from the surface of the water than High line fish (Figure 2c& d). The difference between lines was maintained in the second generation of selection (replicate 1 F_1,163_ = 43.43, *P*<0.0001; replicate 2 F_1,166_ = 246.89, *P*<0.0001). We also detected significant effects of sex in Generation 2 of replicate 1 (*P*<0.0001), and both generations of replicate 2 (*P*<0.0001), where females fed from the surface more quickly than males. The frequency distributions of feeding latency across all generations and replicates are provided in Figure S3.

#### Open field environment

**Figure 3:**
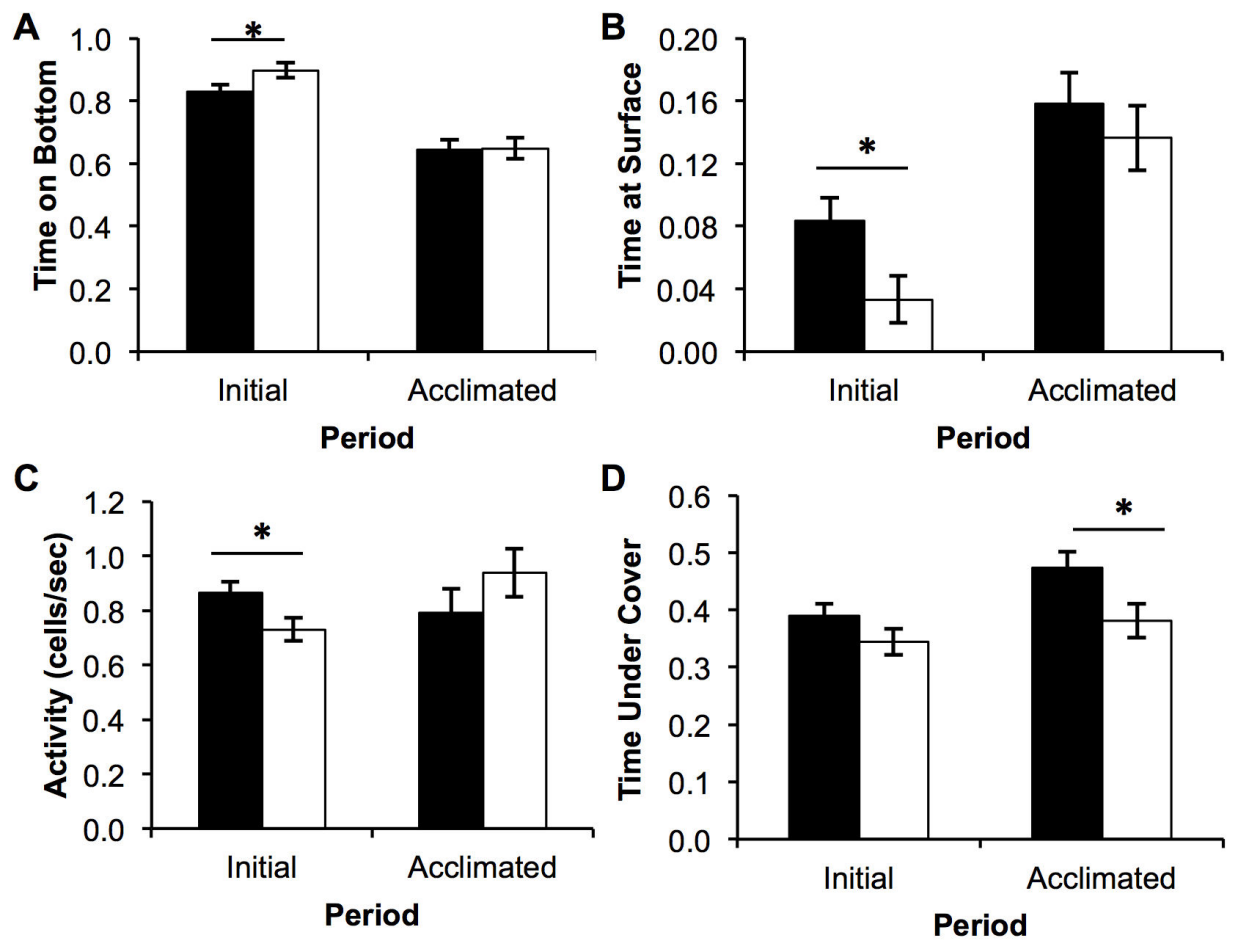
Behavior of second-generation selected lines of zebrafish in an open field environment during two periods. Initial time period is during the introduction to the 19-L aquaria, and Acclimated is after one hour in the aquaria. Proportion of time on the bottom (A) proportion of time at the surface of the tank (B), activity level (C), and proportion of time on the side with cover (D). Filled bars represent means ± SE of the High line, while open bars represent means ±SE of the low line. ‘*’ indicates a significant difference between the lines within a time period using a mixed-model ANOVA.

Several behaviors differed between the lines when fish were placed in the open field aquaria. During the initial four minutes of the assay, Low line fish were more likely than High line fish to freeze motionless (χ^2^ =10.57, df = 1, *P*=0.0011), although there was no difference between the lines in the duration of freezing behavior (F_1,49_=0.03, *P*=0.869). Even when not frozen, Low line fish spent significantly more time on the bottom of the tank, and were less active than High fish (Table 2 Figure 3). After acclimation, the only behavioral difference between the lines was a greater proportion of time spent on the side of the tank with overhead cover by the High fish (Table 2 Figure 3). No significant effects of sex were detected for any of these behaviors (*P*>0.05).

**Table 2:** Mixed model ANOVA for an effect of selected line on zebrafish behavior in a novel environment.

	**Initial**		**Acclimated**	
**Behavior**	**F stat**	**P value**	**F stat**	**P value**
Bottom	4.12	0.0447	0.00	0.9492
Surface	5.77	0.0179	0.60	0.4420
Cover	1.92	0.169	4.90	0.0288
Activity	5.57	0.0199	1.44	0.2328

### Prediction 2: Genetic correlations

The G-matrix analysis of our pedigree data revealed significant genetic correlations among all three behaviors (Table 3). Horizontal position was positively correlated with swim level, where fish spending more time at the front of the tank also spent more time higher in the water column. Horizontal position and swim level were negatively correlated with feeding latency, such that fish at the front or top of the tank also fed more quickly from the surface. Similar significant patterns were detected for phenotypic correlations among all three behaviors (Table 3).

**Table 3:** Genetic and phenotypic correlations and heritabilities (± SE) for three behaviors in zebrafish.

	**Horizontal Position**	**Swim Level**	**Feeding Latency**
Horizontal Position	0.299 ± 0.060	0.626 ± 0.156	0.766 ± 0.110
Swim Level	0.247 ± 0.042	0.260± 0.073	0.851 ± 0.119
Feeding Latency	0.559 ± 0.030	0.396 ± 0.037	0.241 ± 0.065

Heritabilities are provided in the diagonal.

Genetic correlations are provided in the upper off-diagonal.

Phenotypic correlations are provided in the lower off-diagonal.

All estimates were significantly different from zero.

Restricted maximum likelihood analysis of our combined pedigrees in WOMBAT provided a narrow-sense heritability estimate (± standard error) of 0.299 ± 0.06 for horizontal position, 0.260 ± 0.073 for swim level, and 0.241 ± 0.065 for feeding latency (Table 2). All three estimates are significantly different from zero, as determined by constructing 95% confidence intervals.

### Prediction 3: Analysis of G

An eigen analysis of **G** revealed that the three principle components each explain 82.5%, 14.0%, and 3.5% of the total variation. The eigen vector of the first principle component had loadings of 0.569, 0.630, and 0.529 for horizontal position, swim level, and feeding latency respectively. The effective number of dimensions calculated by equation 2 in Kirkpatrick (2009) is ND = 1.21. The angles calculated between the vector of selection gradients, **β**, and **G**max are approximately 57^º^ for the bold line and 115^º^ for the shy line.

## Discussion

We hypothesized that the bold-shy continuum in zebrafish reflects underlying genetic covariance among component behaviors of boldness, predicting that selection on one component would change others. Our first selection experiment confirmed this prediction; High and Low line fish diverged in the selected behavior, as well as other ‘boldness’ behaviors related to place preference and feeding in their ‘home’ tank and activity behavior in a novel environment. We then repeated the experiment with new animals and a new observer, and obtained very similar results in both replicates (even though the behavioral starting points in the parental generations were different, see Figure 1). Across both replicates, analysis of the G matrix revealed the behaviors had significant heritability and were genetically correlated, such that fish that spend more time at the front of the tank near the observer also frequent the top of the tank and begin foraging from the surface more quickly. All of these behaviors have been linked to risk-taking in other studies of fish behavior [21,22,36,37], and we therefore consider the line of fish selected for high horizontal position “Bold” and the line of fish selected for low horizontal position “Shy”. These findings suggest our predictions of the presence of additive genetic variation and genetic correlations among behaviors to be true, and support the hypothesis that evolution along the multivariate bold-shy continuum may be, at least in part, driven by underlying quantitative genetic architecture.

### Mechanisms underlying genetic correlations

There are two classical explanations for our observed genetic correlations – pleiotropy and linkage disequilibrium. Pleiotropy predicts that when we select on a behavior such as horizontal position and observe correlated responses in other behaviors, a single gene affecting multiple behaviors was under selection. Linkage disequilibrium, however, predicts that the genes controlling horizontal position are statistically associated with the genes responsible for the other behaviors. However, these two explanations are not mutually exclusive. Pleiotropy can occur as a result of variation in the genes that govern complex physiological pathways, such as the stress axis. Organisms experiencing stress respond to the stressor and regain homeostasis via physiological and behavioral changes initiated in the hypothalamus-pituitary-interrenal (HPI) axis. Acute behavioral response to stress include alterations to foraging, activity level, space use (place preference), and antipredator behaviors [38]. We have therefore tested third generation fish from these behavioral lines for variation in their stress response, and found significant differences in gene expression [39]. Shy line fish had higher expression of genes regulating production of stress hormones (corticotropin releasing hormone and 11β-hydroxysteroid dehydrogenase) and higher expression of glucocorticoid receptors and brain-derived neurotrophic factor. These genetic differences suggest that variation in stress responsiveness may underlie individual variation in boldness behaviors, and that our fish selected for ‘shy’ behavior were expressing that behavior due to perceived stressors.

### Broader implications for behavioral evolution

One area of study that has observed consistent behavioral evolution along the bold-shy continuum is that of domestication. Wild animals tend to have a shy behavioral type, and evolution in captivity consistently results in a bold behavioral type [40]. This convergent evolutionary pattern has been observed in a diverse array of vertebrate taxa, including birds [41], mammals [6], and fish [25]. Our study showed that behavioral selection can produce divergence in multiple behaviors within one generation in zebrafish, such that fish from the Shy line behave more like wild fish. Our results also match those found in mammalian behavioral selection and domestication studies. Studies of behavioral selection in mammals are much more prevalent than those in fishes, and one of the most well-documented examples involves the domestication and production of foxes. Blue foxes have been shown to respond to behavioral selection for tameness in just three generations, with heritabilities for tameness-related behavioral traits ranging from 0.12 to 0.2 depending on the population and selection program [21]. In addition, a reduced stress response has been documented during domestication, along with the evolution of bold phenotypes (reviewed in 6). These changes have been attributed to an altered fitness landscape, such that fitness in captivity is increased by access to food and mating opportunities from bold behavior, as well as a reduced stress response to common ‘stressors’ [39]. Our results indicate that these consistent emerging behavioral and physiological patterns during domestication may be due to underlying genetic correlations, as opposed to multiple, genetically-independent behavioral responses to a given fitness landscape.

Further, we demonstrated that behavioral selection can produce divergence even in a population (Scientific Hatcheries) that has been maintained in the laboratory for greater than 30 generations. The fact that significant additive variation exists within a strain presumed to be adapted to the laboratory environment [16,17,20] indicates that either these behaviors are not sufficiently linked with fitness in the laboratory to have become fixed, or that variation is being maintained through other mechanisms. The strain used in this study (Scientific Hatcheries) has previously been shown to differ from strains more recently derived from wild populations [16] and shares characteristics with domesticated populations in other fish species [25], such as reduced latency to feed [16], increased food intake and growth rate [16], and increased swim level [15]. Within our experiment, however, we were able to produce a line of Scientific Hatcheries fish behaviorally reminiscent of wild strains. This suggests that conservation programs aimed at limiting domestication may be able to target animals for breeding based on a single behavior, and affect a whole suite of genetically correlated behaviors.

## Conclusion

Our findings confirm our predictions of the presence of additive genetic variation and genetic correlations among boldness behaviors and support the hypothesis that evolution along the multivariate bold-shy continuum may be, at least in part, driven by underlying quantitative genetic architecture. Future work with these lines will provide the opportunity to investigate the role of genetic structure in other axes of personality, such as boldness-aggression syndromes.

## Supporting Information

Figure S1 **Change in frequency distributions (x-axis) of zebrafish horizontal position behavior (y-axis) after selected breeding.**
A) Replicate1 and B) Replicate 2. Generation 0 fish were randomly chosen adult Scientific Hatcheries fish from the Robison lab breeding colony. Generation 1 represents progeny from the first generation of selection (bred from fish shown either as open bars (high line) or black bars (low line) in Generation 1). Similarly, graphs in Generation 2 represent the progeny from the second generation of selection from open or black barred fish in Generation 1.
(TIF)

Figure S2 **Change in frequency distributions (x-axis) of zebrafish swim level behavior (y-axis) after selected breeding based on horizontal position behavior.**
A) Replicate1 and B) Replicate 2. Generation 0 fish were randomly chosen adult Scientific Hatcheries fish from the Robison lab breeding colony. Generation 1 represents progeny from the first generation of selection (bred from fish shown either as open bars (high line) or black bars (low line) in Generation 1). Similarly, graphs in Generation 2 represent the progeny from the second generation of selection from open or black barred fish in Generation 1.
(TIF)

Figure S3 **Change in frequency distributions (x-axis) of zebrafish feeding latency behavior (y-axis) after selected breeding based on horizontal position behavior.**
Data were inverse-transformed (thus higher values reflect more rapid feeding). A) Replicate1 and B) Replicate 2. Generation 0 fish were randomly chosen adult Scientific Hatcheries fish from the Robison lab breeding colony. Generation 1 represents progeny from the first generation of selection (bred from fish shown either as open bars (high line) or black bars (low line) in Generation 1). Similarly, graphs in Generation 2 represent the progeny from the second generation of selection from open or black barred fish in Generation 1.
(TIF)

Table S1 **Sample size for each sex used in each generation of zebrafish breeding.**
(DOCX)
