# Supplementary figures and images for: The Quantitative Genetic Architecture of the Bold-Shy Continuum in Zebrafish, Danio rerio
Source: PLoS One. 2013 Jul 1;8(7):e68828. doi: 10.1371/journal.pone.0068828 (PMC3698077; doi:10.1371/journal.pone.0068828)

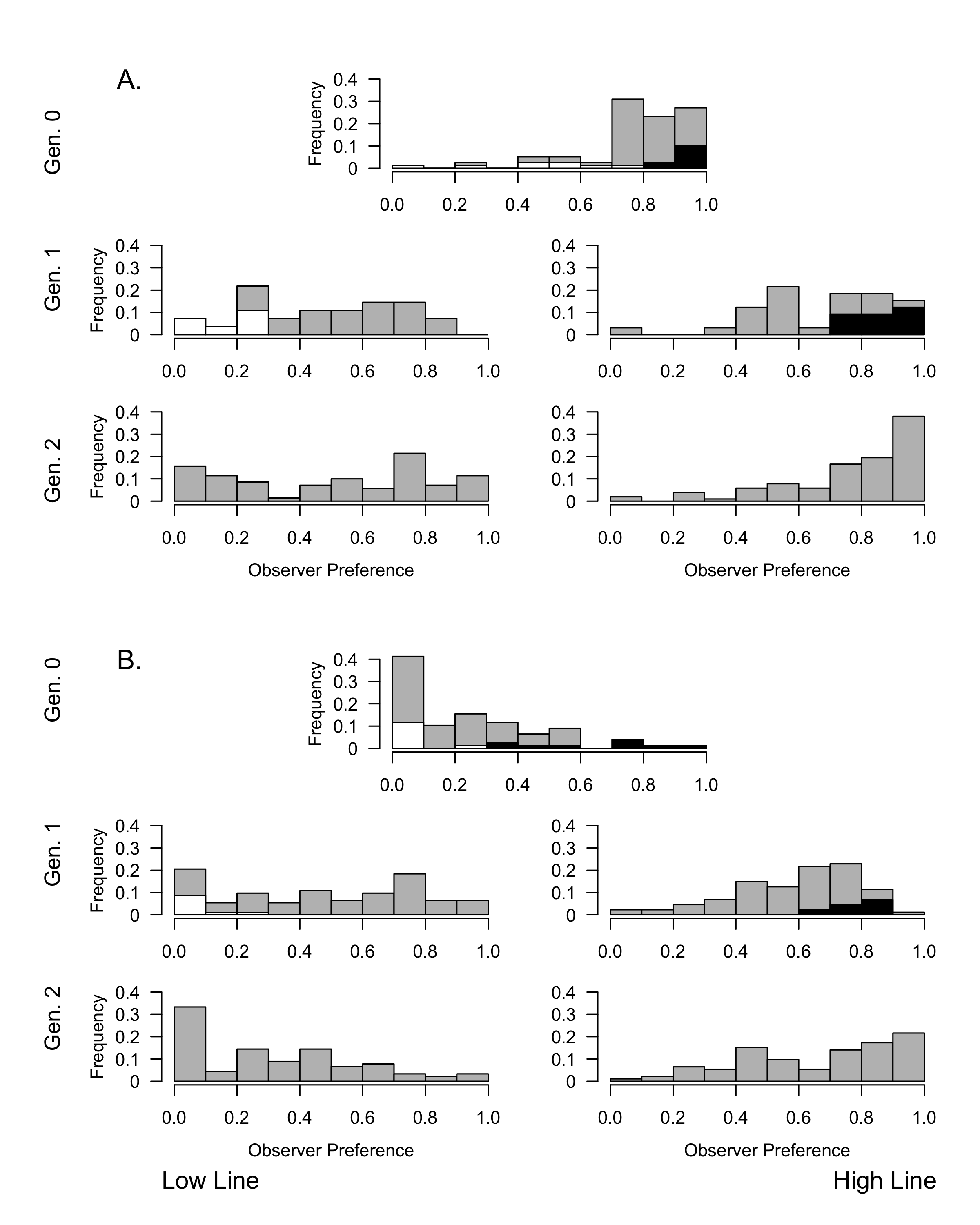

Supplement: Figure S1 — Change in frequency distributions (x-axis) of zebrafish horizontal position behavior (y-axis) after selected breeding. A) Replicate1 and B) Replicate 2. Generation 0 fish were randomly chosen adult Scientific Hatcheries fish from the Robison lab breeding colony. Generation 1 represents progeny from the first generation of selection (bred from fish shown either as open bars (high line) or black bars (low line) in Generation 1). Similarly, graphs in Generation 2 represent the progeny from the second generation of selection from open or black barred fish in Generation 1. (TIF) [file pone.0068828.s001.tif]

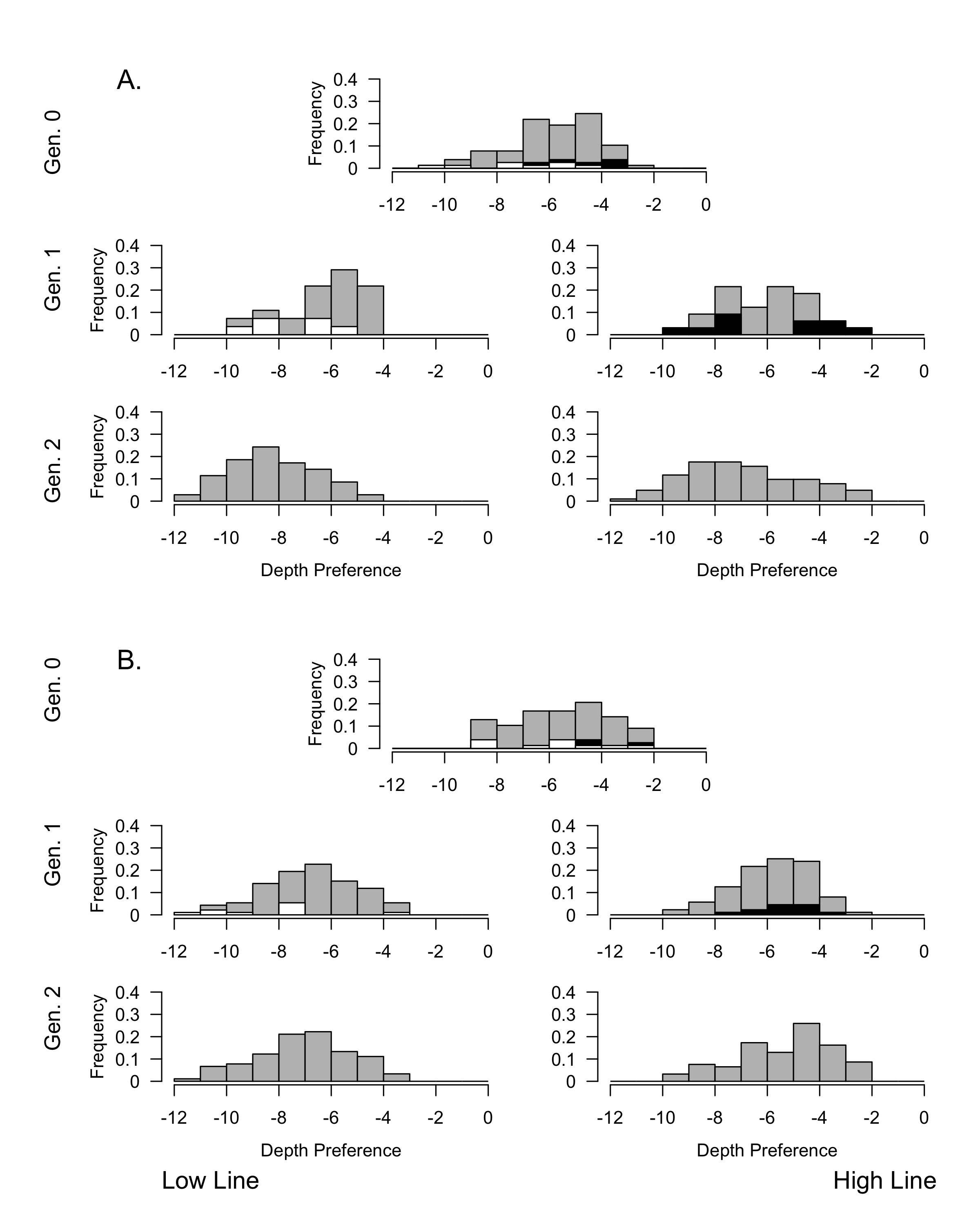

Supplement: Figure S2 — Change in frequency distributions (x-axis) of zebrafish swim level behavior (y-axis) after selected breeding based on horizontal position behavior. A) Replicate1 and B) Replicate 2. Generation 0 fish were randomly chosen adult Scientific Hatcheries fish from the Robison lab breeding colony. Generation 1 represents progeny from the first generation of selection (bred from fish shown either as open bars (high line) or black bars (low line) in Generation 1). Similarly, graphs in Generation 2 represent the progeny from the second generation of selection from open or black barred fish in Generation 1. (TIF) [file pone.0068828.s002.tif]

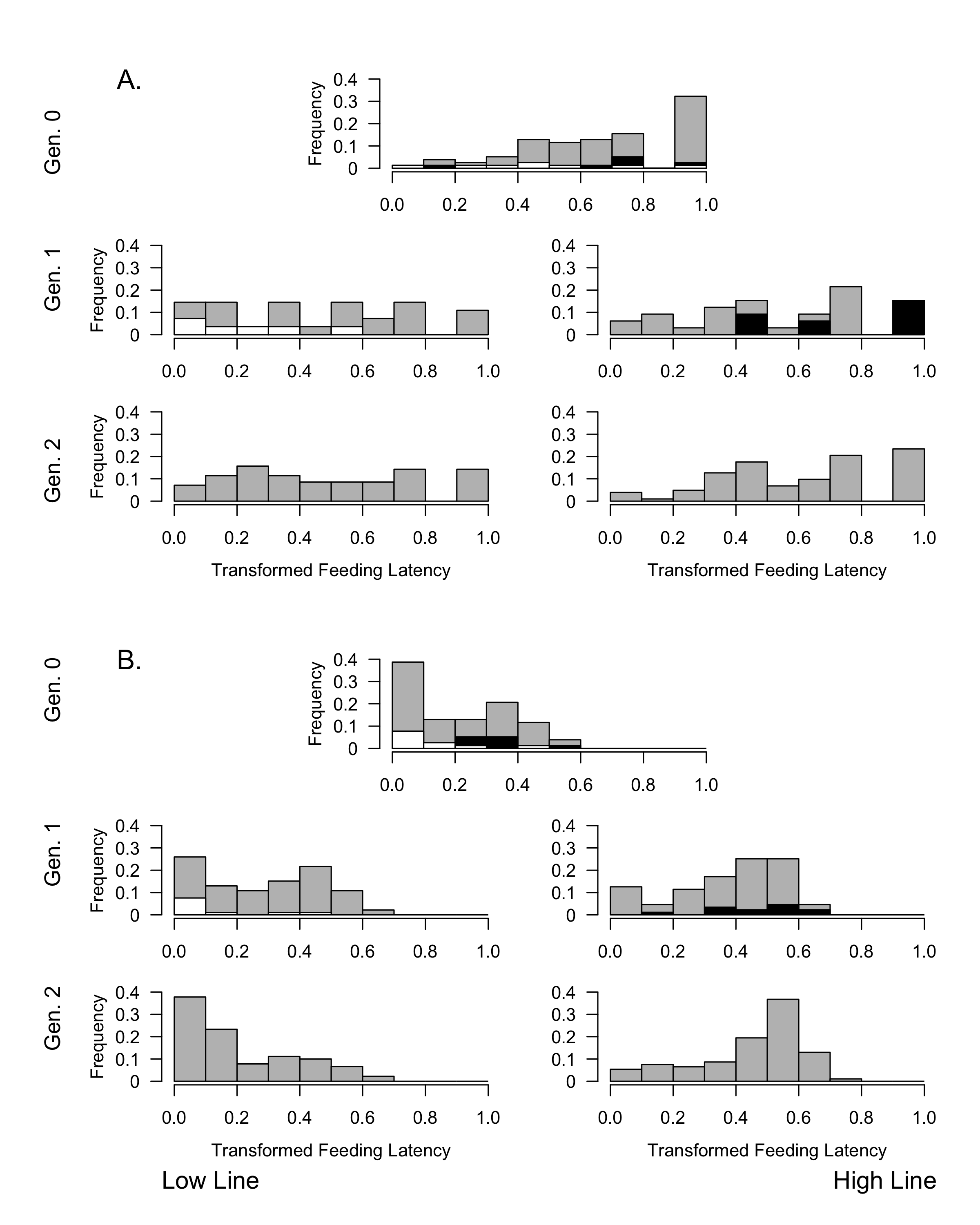

Supplement: Figure S3 — Change in frequency distributions (x-axis) of zebrafish feeding latency behavior (y-axis) after selected breeding based on horizontal position behavior. Data were inverse-transformed (thus higher values reflect more rapid feeding). A) Replicate1 and B) Replicate 2. Generation 0 fish were randomly chosen adult Scientific Hatcheries fish from the Robison lab breeding colony. Generation 1 represents progeny from the first generation of selection (bred from fish shown either as open bars (high line) or black bars (low line) in Generation 1). Similarly, graphs in Generation 2 represent the progeny from the second generation of selection from open or black barred fish in Generation 1. (TIF) [file pone.0068828.s003.tif]
