# Supplementary material for: The Quantitative Genetic Architecture of the Bold-Shy Continuum in Zebrafish, Danio rerio
Source: PLoS One. 2013 Jul 1;8(7):e68828. doi: 10.1371/journal.pone.0068828 (PMC3698077; doi:10.1371/journal.pone.0068828)
Supplement: Table S1 — Sample size for each sex used in each generation of zebrafish breeding. (DOCX) [file pone.0068828.s004.docx]

**Supplemental Table 1.** **Sample size for each sex used in each generation of zebrafish breeding.**

| - - 1. **Replicate** | - - 1. **Generation** | **Line** | - - 1. **Male** | - - 1. **Female** |
| --- | --- | --- | --- | --- |
| - - 1. 1 | - - 1. 0 | - - 1. High | - - 1. 5 | - - 1. 5 |
|  |  | - - 1. Low | - - 1. 5 | - - 1. 5 |
|  | - - 1. 1 | - - 1. High | - - 1. 11 | - - 1. 20 |
|  |  | - - 1. Low | - - 1. 4 | - - 1. 23 |
|  | - - 1. 2 | - - 1. High | - - 1. 59 | - - 1. 44 |
|  |  | - - 1. Low | - - 1. 35 | - - 1. 35 |
| - - 1. 2 | - - 1. 0 | - - 1. High | - - 1. 5 | - - 1. 5 |
|  |  | - - 1. Low | - - 1. 5 | - - 1. 5 |
|  | - - 1. 1 | - - 1. High | - - 1. 61 | - - 1. 27 |
|  |  | - - 1. Low | - - 1. 44 | - - 1. 48 |
|  | - - 1. 2 | - - 1. High | - - 1. 36 | - - 1. 38 |
|  |  | - - 1. Low | - - 1. 46 | - - 1. 59 |
